# P-2197. Impact of ventilation on transmission risk and reproduction number of viruses in South India: implications for climate change and pandemic preparedness

**DOI:** 10.1093/ofid/ofaf695.2360

**Published:** 2026-01-11

**Authors:** Palak Shah, Brady Sack, Abdul Basith, Madolyn Dauphinais, Komal Jain, Maria Florencia Martins, Sierra Wallace, Subitha Lakshminarayanan, Chelsie Cintron, Sadhana Subramanian, Apratim Sahay, Kobto Koura, Ralph P Brooks, Sheela Shenoi, Pranay Sinha, Palanivel Chinnakali, Lauren Pischel

**Affiliations:** Boston University Chobanian and Avedisian School of Medicine, Boston, Massachusetts; University of Virginia School of Medicine, Charlottesville, Virginia; Jawaharlal Institute of Postgraduate Medical Education and Research, Puducherry, Puducherry, India; Boson Medical Center, Boston, Massachusetts; Jawaharlal Institute of Postgraduate Medical Education and Research, Puducherry, Puducherry, India; Boston University Medical Center, Boston, Massachusetts; Boston University Medical Center, Boston, Massachusetts; Jawaharlal Institute of Postgraduate Medical Education and Research, Puducherry, Puducherry, India; Boston University Medical Center, Boston, Massachusetts; Jawaharlal Institute of Postgraduate Medical Education and Research, Puducherry, Puducherry, India; Johns Hopkins University, Baltimore, Maryland; International Union Against Tuberculosis and Lung Disease, Paris, Ile-de-France, France; Yale School of Medicine - AIDS Program, New Haven, Connecticut; Yale University, New Haven, CT; Boston University, Boston, Massachusetts; Jawaharlal Institute of Postgraduate Medical Education and Research, Puducherry, Puducherry, India; Yale School of Medicine, New Haven, Connecticut

## Abstract

**Background:**

Rising global temperatures are predicted to increase the time individuals spend in under-ventilated indoor spaces, especially in tropical and subtropical regions, and to enhance the transmission risk of respiratory pathogens. We studied the impact of ventilation on the transmission risk and basic reproductive number (R_0_) of common respiratory viruses that have pandemic potential, namely SARS-CoV-2 and influenza, in Puducherry, India.

**Figure:**
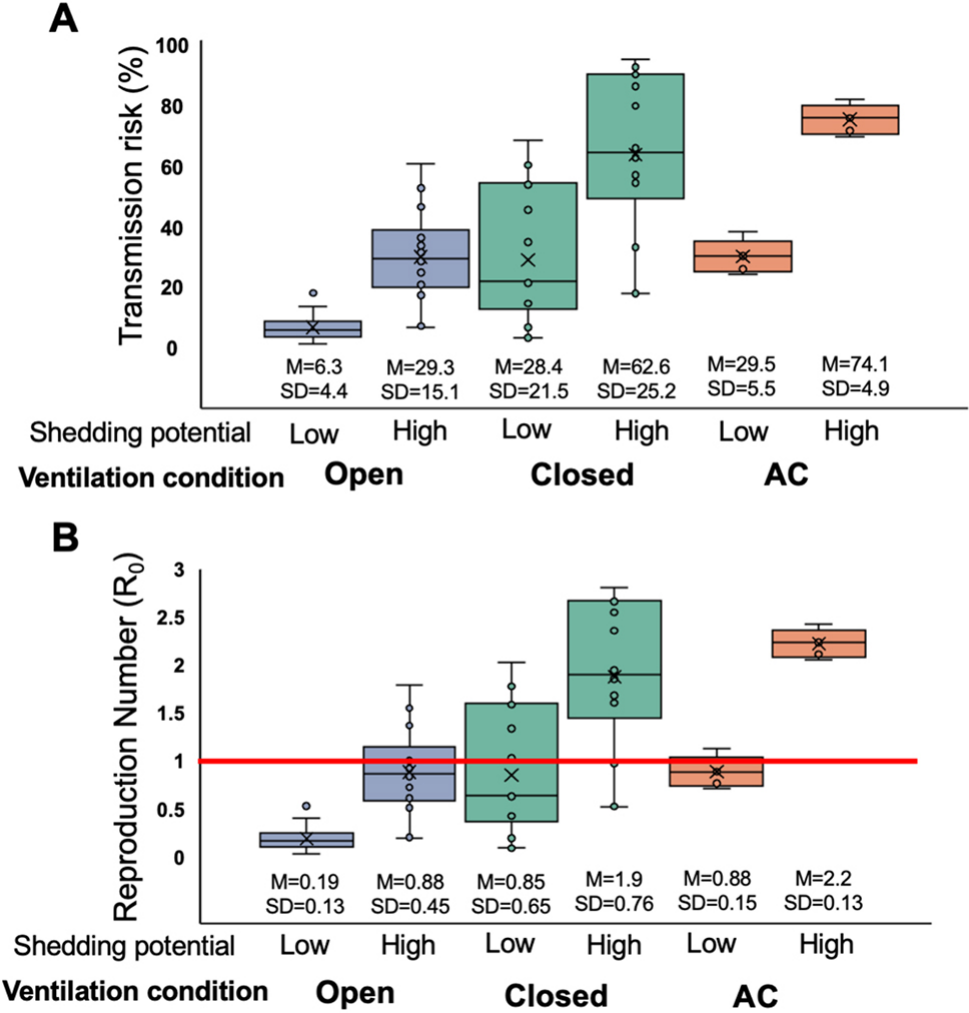
Transmission risk and basic reproductive number (R_0_) of SARS-CoV-2 under different ventilation conditions and viral shedding potentials. 1A) Box plots of transmission risk using base estimate of q= 28.0 for high viral shedding potential and q= 4.0 for low viral shedding potential, across three ventilation conditions tested. Solid line at top denotes Wilcoxon rank sum test comparing closed and open conditions for each viral shedding potential; M denotes mean, SD denotes standard deviation. 1B) Box plots of R_0_ for each ventilation condition and viral shedding potential. Bright red line denotes R_0_ of 1.

**Figure:**
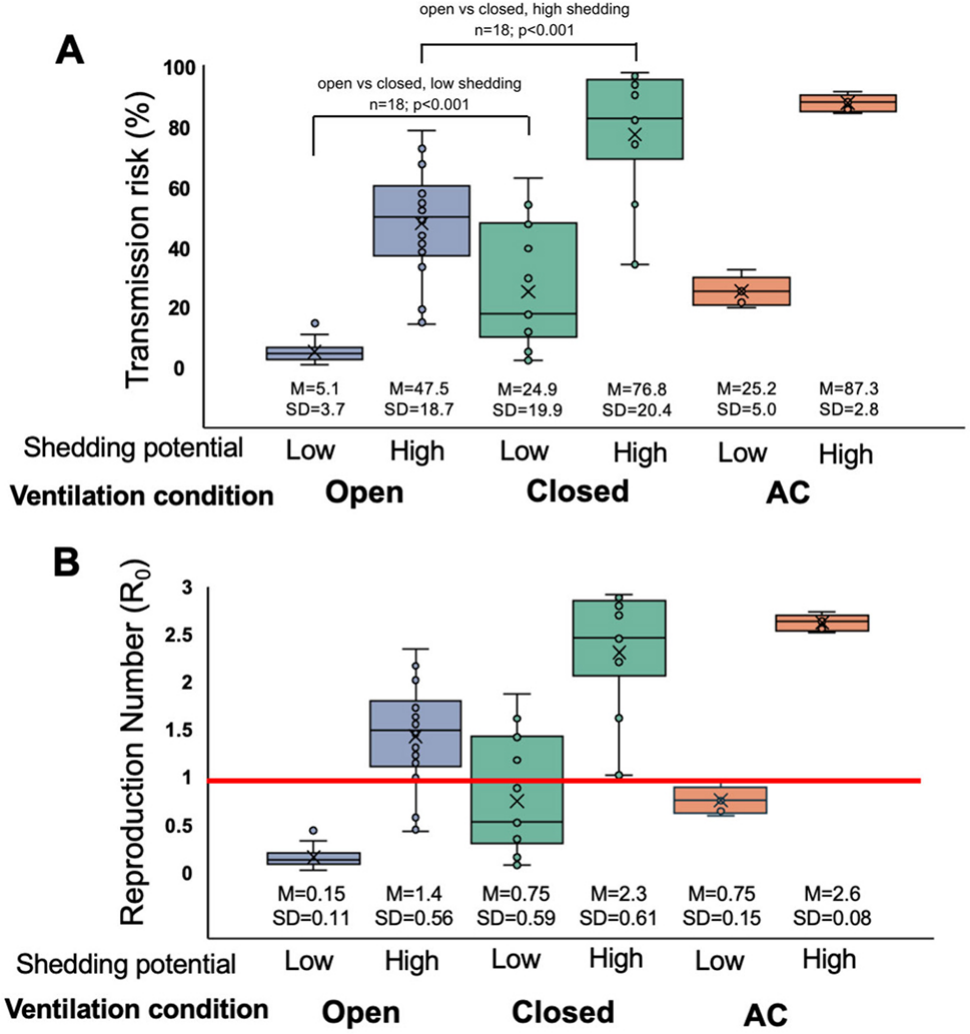
Transmission risk and basic reproductive number (R_0_) of influenza under different ventilation conditions and viral shedding potentials 2A) Box plots of transmission risk using base estimate of q= 68.0 for high viral shedding potential and q= 3.2 for low viral shedding potential, across three ventilation conditions tested. Solid line at top denotes Wilcoxon rank sum test comparing closed and open conditions for each viral shedding potential; M denotes mean, SD denotes standard deviation. 2B) Box plots of R_0_ for each ventilation condition and viral shedding potential. Bright red line denotes R_0_ of 1.

**Methods:**

We measured ventilation in air changes per hour in homes and healthcare offices using a carbon-dioxide decay technique. We applied the Wells-Riley equation to estimate the transmission risk and R_0_ of SARS-CoV-2 and influenza in these settings, as well as under different ventilation conditions and viral shedding levels.

**Results:**

We conducted 45 ventilation measurements across 13 homes and 7 offices; four of five air conditioning (AC) measurements were in offices. In the closed condition (doors/windows closed, fans off), mean SARS-CoV-2 transmission risk was high for high virus shedders (62.6%, SD 25.2%) and lower for low shedders (28.4%, SD 21.5%) (Figure 1A). Risk decreased significantly in the open condition (doors/windows open, fan on) for both high (29.3%, SD 15.1%; p< 0.001) and low shedders (6.3%, SD 4.4%; p< 0.001). Under AC, transmission risk remained similar to the closed condition for low shedders but was highest for high shedders (74.1%, SD 4.9%). For high shedders, R₀ equaled or exceeded 2 in both the closed (1.9, SD 0.76) and AC (2.2, SD 0.13) conditions, but stayed below 1 for low shedders across all ventilation scenarios (Figure 1B).

Similarly, for influenza, transmission risk was high in the closed condition for high virus shedders (76.8%, SD 20.4%) and decreased significantly in the open condition (47.5%, SD 18.7%; p< 0.001) (Figure 2A). For low shedders, transmission risk remained low in both the closed (24.9%, SD 19.9%) and AC (25.2%, SD 5.0%) conditions. The R₀ for influenza exceeded 2 in the closed and AC conditions for high shedders (Figure 2B).

**Conclusion:**

The transmission risk of respiratory viruses in homes and healthcare spaces is high, particularly with the use of AC. Under-ventilation increases R_0_ above 1 among high virus shedders.

**Disclosures:**

Ralph P. Brooks, MS, Merck: Stocks/Bonds (Public Company) Sheela Shenoi, MD MPH, Merck Pharmaceuticals: My spouse worked for Merck 1997-2007 and retains company stock in his retirement account. There is no conflict of interest with this work. Lauren Pischel, MD, Auxa Health: Advisor/Consultant

